# Artificial intelligence in global health equity: an evaluation and discussion on the application of ChatGPT, in the Chinese National Medical Licensing Examination

**DOI:** 10.3389/fmed.2023.1237432

**Published:** 2023-10-19

**Authors:** Wenting Tong, Yongfu Guan, Jinping Chen, Xixuan Huang, Yuting Zhong, Changrong Zhang, Hui Zhang

**Affiliations:** ^1^Department of Pharmacy, Gannan Healthcare Vocational College, Ganzhou, Jiangxi, China; ^2^Department of Rehabilitation and Elderly Care, Gannan Healthcare Vocational College, Ganzhou, Jiangxi, China; ^3^Department of Mathematics, Xiamen University, Xiamen, Fujian, China; ^4^Department of Anesthesiology, Gannan Medical University, Jiangxi, China; ^5^Department of Chinese Medicine, Affiliated Hospital of Qinghai University, Xining, Qinghai, China; ^6^Chair of Endocrinology and Medical Sexology (ENDOSEX), Department of Experimental Medicine, University of Rome Tor Vergata, Rome, Italy

**Keywords:** global healthcare, equity, artificial intelligence, ChatGPT, language bias

## Abstract

**Background:**

The demand for healthcare is increasing globally, with notable disparities in access to resources, especially in Asia, Africa, and Latin America. The rapid development of Artificial Intelligence (AI) technologies, such as OpenAI’s ChatGPT, has shown promise in revolutionizing healthcare. However, potential challenges, including the need for specialized medical training, privacy concerns, and language bias, require attention.

**Methods:**

To assess the applicability and limitations of ChatGPT in Chinese and English settings, we designed an experiment evaluating its performance in the 2022 National Medical Licensing Examination (NMLE) in China. For a standardized evaluation, we used the comprehensive written part of the NMLE, translated into English by a bilingual expert. All questions were input into ChatGPT, which provided answers and reasons for choosing them. Responses were evaluated for “information quality” using the Likert scale.

**Results:**

ChatGPT demonstrated a correct response rate of 81.25% for Chinese and 86.25% for English questions. Logistic regression analysis showed that neither the difficulty nor the subject matter of the questions was a significant factor in AI errors. The Brier Scores, indicating predictive accuracy, were 0.19 for Chinese and 0.14 for English, indicating good predictive performance. The average quality score for English responses was excellent (4.43 point), slightly higher than for Chinese (4.34 point).

**Conclusion:**

While AI language models like ChatGPT show promise for global healthcare, language bias is a key challenge. Ensuring that such technologies are robustly trained and sensitive to multiple languages and cultures is vital. Further research into AI’s role in healthcare, particularly in areas with limited resources, is warranted.

## Introduction

**Figure 1:**
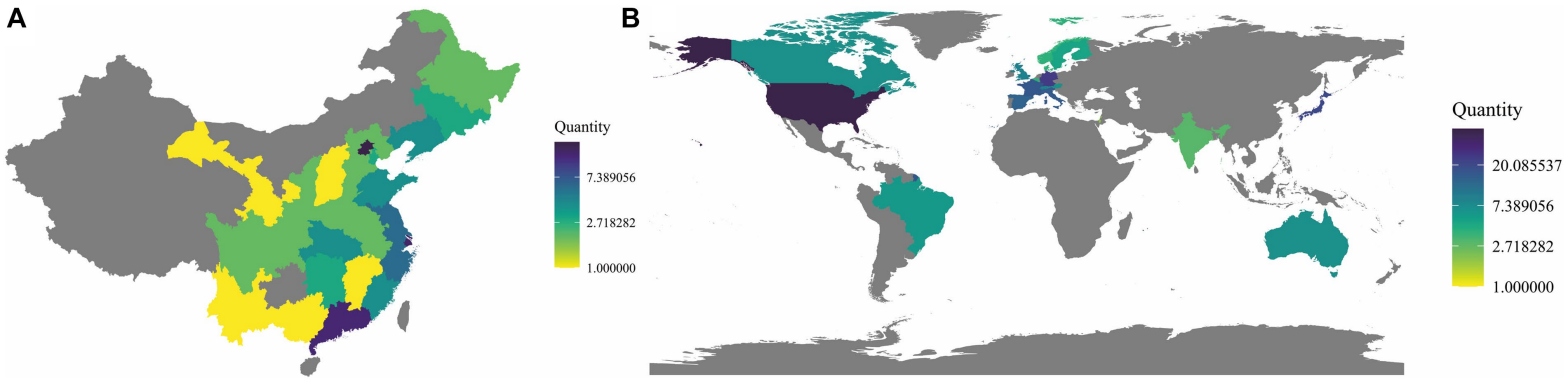
Distribution of top-tier hospitals in China and around the world. **(A)** Distribution of top-tier hospitals in China. The 2021 hospital rankings developed by the Institute of Hospital Management at Fudan University (fudanmed.com) (the data from Taiwan, Hong Kong, and Macau are not included in the statistical scope). **(B)** Distribution of the top 250 hospitals worldwide. The 2022 hospital rankings compiled by Newsweek (www.newsweek.com).

In a global context, the demand for healthcare is continuously escalating, yet the distribution of medical resources is uneven across different regions, particularly in parts of Asia, Africa, and Latin America (Figure 1) (1). This phenomenon was especially pronounced during the COVID-19 pandemic (2, 3). Numerous factors contribute to this situation, including historical legacies, cultural backgrounds, political systems, technological infrastructures, and economic development (4, 5). Despite considerable efforts made by various international organizations, charitable institutions, governments, and non-governmental organizations through fiscal aid, technological support, and human resource training to mitigate this inequality, achieving global equity in the distribution of healthcare resources still necessitates greater investment and cooperation (3, 6).

Meanwhile, the rapid development of artificial intelligence (AI) technologies, such as OpenAI’s GPT-4 model, ChatGPT, have shown the potential to disrupt established practices in the medical field (7, 8). As a robust language model, ChatGPT shows promising accuracy in many tasks and broad applicability potential by understanding and generating human languages. Its efficacy has been proven in the United States Medical Licensing Examination (9). AI-based medical innovations pose new challenges to physicians, particularly internists (10), but they also bring unique opportunities for areas with scarce medical resources. OpenAI’s ChatGPT project opens theoretical possibilities for global healthcare equality and has tangible potential (11).

However, this is not to say that ChatGPT is without its challenges. The main challenges lie in two aspects: firstly, ChatGPT requires specialized training in the medical field to offer effective diagnostic and treatment advice (12, 13). Secondly, the issue of privacy is an important factor to consider (14). The global handling of private data has provoked considerable debate and even led some countries to temporarily ban the use of ChatGPT (15–18). It’s noteworthy that solutions to these challenges are currently being implemented. Regarding medical training, collaboration with medical experts is essential, routinely assessing and validating its diagnostic recommendations to ensure accuracy and reliability. As for privacy concerns, stricter data protection policies and regulations are needed to ensure the lawful use of data and protection of users’ privacy rights.

However, given that the training of ChatGPT is based on Internet data, and considering the massive disparity in the volume of Internet data between other languages, such as Chinese, and English (19), we have reason to suspect the existence of language bias (20). Therefore, although ChatGPT performs well in English-speaking environments, we still need to conduct comparative research based on other languages.

To further explore this issue, we have designed an experiment aimed at evaluating ChatGPT’s applicability and limitations in taking the Chinese Medical Licensing Examination in both English and Chinese contexts. Through this experiment, we hope to gain a deeper understanding of ChatGPT’s potential and challenges in the medical field, and the bias in different language environments provide references for future research and development in the field of medical artificial intelligence.

## Methodology

This study is an experimental, quantitative research. To ensure standardization of evaluation and to prevent ChatGPT from directly utilizing its pre-existing training database (for instance, the cut-off date for the training data of ChatGPT 4.0 is September 2021), we opted to use the comprehensive written section of the 2022 National Medical Licensing Examination (NMLE) as our evaluation standard. The relevant questions were provided by Beijing Medical Examination Assistance Technology Co., Ltd. For each question included in the study, we translated the Chinese version into English, guided by an expert (JL L) proficient in English and possessing a medical professional background, thereby generating the corresponding English version.

The question bank consists of 600 questions, each worth 1 point, totaling 600 points. Scoring 60% of the total points is considered passing. The test is divided into four parts, each covering:

Basic Medical Comprehensive: Exam topics include Physiology, Biochemistry, Pathology, Pharmacology, Medical Microbiology, Medical Immunology, Anatomy, and Pathophysiology.

Medical Humanities Comprehensive: Topics are Health Regulations, Medical Psychology, and Medical Ethics.

Clinical Medicine Comprehensive: Exam topics are Internal Medicine (including Infectious Diseases), Surgery, Obstetrics and Gynecology, Pediatrics, Neurology, and Psychiatry.

Preventive Medicine Comprehensive: The subject is Preventive Medicine.

Each part contains various question types, namely A1, A2, A3, A4, and B1:

A1 Type (Single Sentence Best Choice Question): Each question consists of 1 stem and 5 optional answers. Only 1 is the best choice, while the other 4 are distractors. These distractors might be entirely incorrect or partially correct.

A2 Type (Case Summary Best Choice Question): The structure of the question includes 1 brief medical history as the stem, followed by 5 optional answers, with only one being the best choice.

B1 Type (Standard Matching Question): The question starts with 5 optional answers. After these options, at least 2 questions are given. Test takers are required to choose one closely related answer for each question. In a set of questions, each optional answer can be used once, several times, or not at all.

A3 Type (Case Group Best Choice Question): The structure begins by describing a clinical scenario centered on a patient. Then, 2–3 related questions are given. Each question is related to the initial clinical situation but tests different points, and the questions are independent of each other.

A4 Type (Case Sequence Best Choice Question): The structure starts by narrating a clinical situation centered around a single patient or family, followed by 3–6 related questions. As the case unfolds, new information can be progressively added. Sometimes, some minor or hypothetical information is provided, which may not necessarily be related to the specific patient described in the case.

The sample size was determined according to a formula: $n=z_{\frac{a}{2}}\sqrt{2p\left(1-P\right)}+z\beta \sqrt{\frac{\left[P_{1}\left(1-P_{1}\right)+P_{2}\left(1-P_{2}\right)\right]}{{\left(P_{1}-P_{2}\right)}^{2}}}$ , where n is the sample size per group, Z_(α/2) is the Z score of α/2 (we set α = 0.05, hence Z_(α/2) ≈ 1.96), Z_β is the Z score of β (we set β = 0.20, hence Z_β ≈ 0.84), P1 and P2 are the expected accuracy rates of the Chinese version and the English version, respectively. We set P1 = 0.8, P2 = 0.85. Based on these parameters, we calculated that each version required 77 questions, totaling 154. For further analysis, we eventually randomly selected 160 questions as samples, and the standard answers were provided by experts (XC T, Q H) with extensive clinical experience and practice licenses.

On May 3rd and 4th, 2023, each question was entered respectively, to avoid interference from the English version to the Chinese version, we test the Chinese and English in separate dialogue boxes. The method of inquiry is to directly copy the question and instruct ChatGPT 4.0 to answer and explain. No additional explanations will be provided beyond this. All questions are asked only once. Furthermore, the scoring method adopted by China’s NMLE is straightforward; every question is worth one point, with no deductions for wrong answers. Therefore, when calculating the accuracy rate, we follow the official scoring method, which is a simple addition. These answers were rated for “information quality” by three evaluators (JP C, YT Z, CR Z) who hold medical practice licenses, using the Likert scale, with ratings ranging from “very poor” to “excellent.” All answer scores were converted to a scale of 1–5, with 5 indicating “excellent.” We adopted a strategy of combined ratings, i.e., merging the scores of the three experts, and calculating the average score of the evaluators for each research discussion. In the absence of an absolute standard, the assessment is subjective, so the average score reflects consensus among evaluators, while discrepancies (or inherent ambiguities and uncertainties) are reflected in the variance of the scores. We will compare the average quality of the answers in both versions. Depending on the data distribution, we will use a t-test or Mann–Whitney U test to compare the average quality of the answers in the two versions.

To investigate factors that might affect the accuracy of ChatGPT 4’s responses, we performed a binary logistic regression analysis to evaluate whether the difficulty of the questions or the disciplines to which they belong were associated with AI’s incorrect answers. We classified questions by discipline and asked three junior clinicians who scored average (60% of total), good (70%), and excellent (80%) in the practice exams to rate the difficulty of the questions using the Likert scale, where the highest difficulty is 5 points and the lowest difficulty is 1 point.

We employed the Brier Score to evaluate ChatGPT 4’s diagnostic efficiency in both language versions. The Brier Score is a method to measure the accuracy of diagnostic prediction. It is the mean of the squared differences between the predicted probability (p) and the actual outcome (o) {i.e., Brier Score = mean[(p-o)^2^]}. A Brier Score between 0 and 1, with a score closer to 0 indicating higher prediction accuracy. We calculated a Brier Score for each possible diagnosis and took the average to obtain ChatGPT’s overall prediction accuracy in different linguistic environments.

All statistical analyses were performed using the R software package (version 4.2.2).

In our research, all utilized information was obtained from public databases, and the involved questions did not contain any identifiable personal information. Consequently, as per the relevant ethical regulations, this study does not involve the handling of personal privacy and confidential information and is thus exempt from a specific ethical review.

## Results

**Figure 2:**
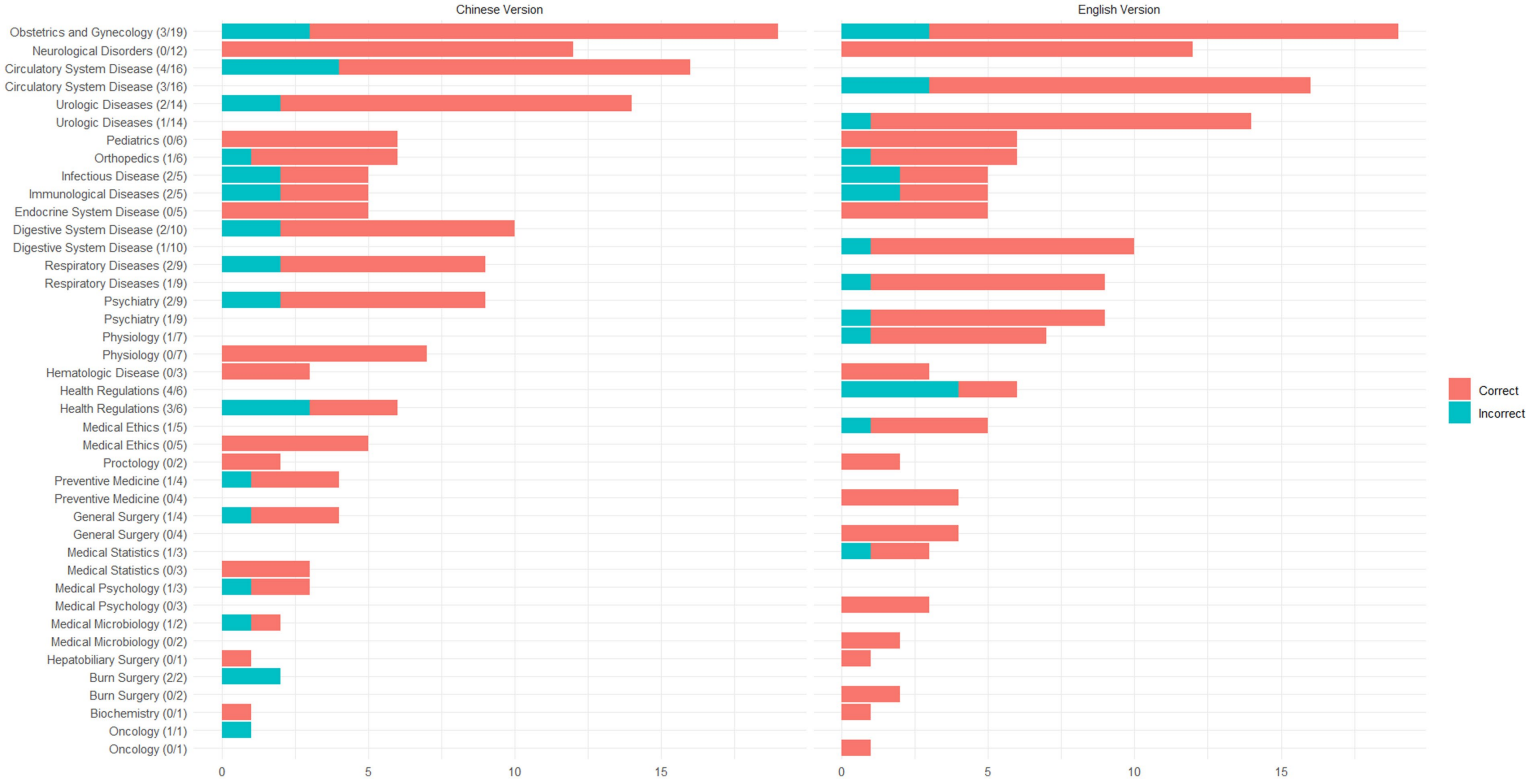
Distribution of questions and accuracy rates across categories.

In this study, a total of 160 questions were included, covering 26 categories such as Psychiatry, Health Regulations, Urologic Diseases, and Biochemistry. For these questions, ChatGPT demonstrated an accuracy rate of 81.25% (95% CI, 74.32–86.98%) in responding to the Chinese versions and 86.25% (95% CI, 79.93–91.18%) in responding to the English versions (Fisher’s exact test, OR: 2.99, 95% CI, 0.97–8.77, *p* = 0.04). This suggests that ChatGPT shows higher accuracy in answering medical questions in English compared to Chinese. The number of questions and incorrect answers for each category are shown in Figure 2.

We conducted a thorough analysis of ChatGPT’s responses to questions in both Chinese and English. In the Chinese version, the main reasons for errors were: influence of specific regional policies and regulations in China (16.7%, 5/30), unclear question descriptions or vague answers (23.3%, 7/30), incomplete analysis (40%, 12/30), and other undefined factors (20%, 6/30). In the English version, the primary causes of errors were: unclear question descriptions (31.8%, 7/22), influence of specific regional policies and regulations in China (27.3%, 6/22), insufficient grasp of information (22.7%, 5/22), and other undefined factors (18.2%, 4/22) (Supplementary Appendix 1).

Three medical students with different performances in the medical licensing examination rated the difficulty of the 160 questions. The results revealed that the relative difficulty ratings were 2.41 ± 0.53, 3.38 ± 0.57, and 4.37 ± 0.57, respectively. Upon conducting binary logistic regression analysis, it was found that neither the difficulty nor the category of the question was a significant factor leading to errors in AI responses (all *p* > 0.05). Moreover, evaluations using the Brier Score showed a score of 0.19 for the Chinese version and 0.14 for the English version, indicating that the AI demonstrated good predictive performance in dealing with both Chinese and English questions (Table 1).

**Table 1:** Logistic regression results and brier scores for Chinese and English versions.

	Estimate	SE	*z*-value	Pr(>|z|)	Brier score
Chinese version					0.19
(Intercept)	0.15	1.70	0.09	0.93	
High level	−0.63	0.90	−0.70	0.49	
Medium level	−0.82	1.15	−0.71	0.48	
Low level	0.53	0.87	0.61	0.54	
Category	0.02	0.03	0.65	0.51	
English version					0.14
(Intercept)	0.02	1.90	0.01	0.99	
High level	0.20	0.94	0.22	0.83	
Medium level	0.30	1.17	0.26	0.80	
Low level	−0.78	0.93	−0.84	0.40	
Category	0.00	0.03	−0.01	0.99	

**Figure 3:**
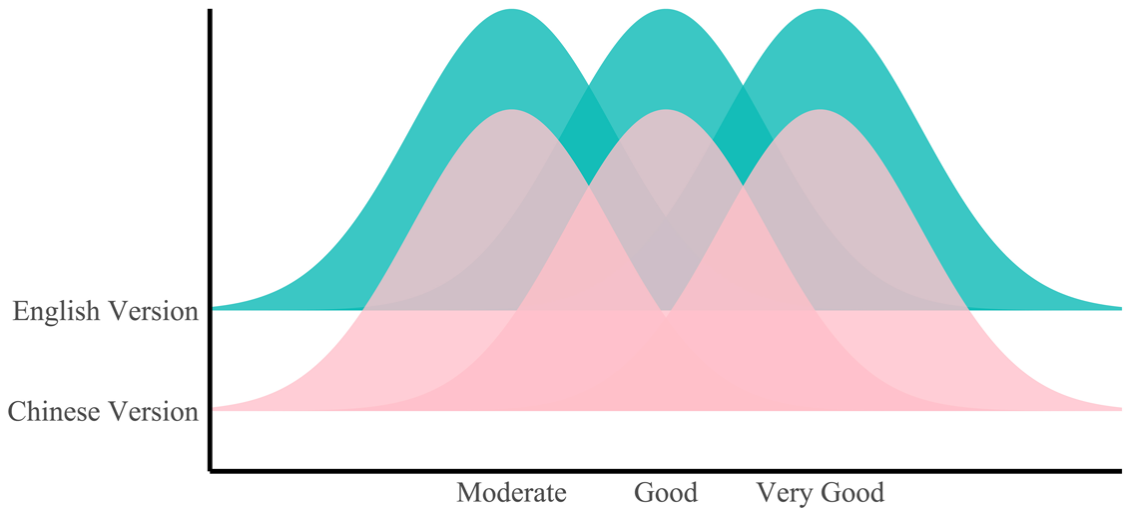
Ridge plot of quality ratings for the Chinese and English versions.

In terms of response quality, the English version exhibited superior performance, achieving an excellent mean rating of 4.43 (95% confidence interval, 4.37–4.48), outperforming the equally excellent rating of the Chinese version (4.34, 95% confidence interval, 4.29–4.4) (*W* = 107,499, *p* = 0.04628) (Figure 3). Specifically, the proportion of responses in the Chinese version that fell below excellence stood at 9.79% (95% confidence interval, 7.13–12.45%), a ratio higher than the 7.92% (95% confidence interval, 5.50–10.33%) observed in the English version.

## Discussion

The persistent, significant issue of uneven global medical resource distribution has long plagued human societies (1). In many parts of the globe, particularly in regions of Asia, Africa, and Latin America, an acute mismatch exists between healthcare demands and resource supplies due to a complex interplay of factors such as historical residues, cultural contexts, political regimes, deficiencies in technological infrastructure, and disparate levels of economic development (11). The COVID-19 pandemic has further accentuated this global health crisis (2, 3), with the international community striving to find effective solutions. Against this backdrop, artificial intelligence (AI) technologies, notably OpenAI’s GPT-4 model—ChatGPT, have emerged as a vital tool in addressing the inequality in healthcare resource distribution (21, 22).

The aim of this study is to scrutinize and discuss the application potential and limitations of ChatGPT in the context of the NMLE in both English and Chinese environments. The NMLE serves as an essential means of comprehensively evaluating the professional competencies and practical skills of medical school graduates or individuals working in medical institutions (23). In prior research, studies have investigated the performance of CHATGPT in the United States medical licensing examination. Their findings align with ours, demonstrating commendable results (24–26). Moreover, due to concerns about language bias (27), After utilizing ChatGPT to assess the English version of the Chinese medical licensing examination, we further explored the differences in ChatGPT’s handling of both the English and Chinese versions of the NMLE. Our findings suggest that ChatGPT performs more robustly when addressing English medical queries compared to its Chinese counterparts, indicating that, despite its considerable application potential, challenges remain when dealing with questions in non-English settings. This issue not only bears relevance to technological advancements but also directly affects our approach toward leveraging such tools to mitigate global healthcare resource imbalances.

Further analysis reveals that the difficulty and type of questions do not significantly impact AI performance, suggesting a relatively stable performance of ChatGPT when dealing with complex or specific types of questions. However, it is imperative to consider that, despite the varying levels of difficulty, the questions involved in this study pertain merely to entry-level examinations in the medical profession. When compared to intricate clinical scenarios, these questions still appear relatively straightforward. Furthermore, the results of this study do not imply that we can overlook the challenges ChatGPT might encounter when learning and adapting to various types of questions. To offer effective medical diagnostic and treatment suggestions, ChatGPT requires specialized training in the medical field, necessitating the formulation of effective training strategies to enhance ChatGPT’s understanding and processing capabilities for different types of questions (28).

Moreover, our results show that ChatGPT exhibits solid predictive performance when handling both English and Chinese queries. Yet, akin to accuracy, the quality of answers in English surpasses those in Chinese. This likely mirrors ChatGPT’s linguistic advantage when dealing with English questions and the challenges arising from language and cultural discrepancies when addressing non-English queries (29). For instance, difficulties in language comprehension, regulations and interpretation errors arising from cultural differences are pressing issues requiring attention (27, 30, 31). Consequently, we suggest AI tool developers need to gain a deeper understanding and appreciation of non-English cultures and languages to tailor and optimize AI tools more effectively. Simultaneously, more robust assessment and regulatory mechanisms need to be established to prevent the usage of AI tools from inciting new unfairness and discrimination (28).

In the application of artificial intelligence in healthcare, ethical considerations should always be central, especially when AI technologies begin to involve medical diagnostics and treatment decision-making (32–34). Promoting unapproved treatments or tolerating unethical medical procedures could potentially provoke a raft of ethical issues. First and foremost, patient safety is at the core of medical care. When introducing AI technologies, such as ChatGPT, to provide medical advice to patients, it is imperative to ensure that they are based on reliable medical data and practices, ensuring that the recommendations given are accurate and safe (35). Secondly, ethics and human rights occupy a central position in the design and application of AI. This means that when using these technologies, patients’ rights and privacy should be respected, ensuring transparency and accountability (36, 37). It’s worth noting that, although AI might excel in certain tasks, it cannot fully replace human doctors. The strengths of technology and doctors should complement each other (38). For instance, AI can process vast amounts of data and provide preliminary suggestions, while doctors can use their experience and intuition for the final diagnosis and treatment decisions (22, 39). With the introduction of new technologies, maintaining transparency and trust among doctors, patients, and medical institutions becomes essential. This also demands that all stakeholders understand how AI works and its inherent limitations (40). In its current design, GPT-4 does not explicitly incorporate ethical guidelines (34, 41–44), which could prove problematic under certain circumstances. Hence, future research and development should consider integrating ethical norms into AI models to ensure their safe and compliant application in the medical field.

In conclusion, our research highlights the potential application of AI in healthcare, but a substantial amount of research and experimentation is still required to truly integrate GPT-4 or other AI technologies into medical services. This includes model optimization, environment adaptation, ethical and legal issue handling, and the development of culturally sensitive AI models for non-English settings. We look forward to witnessing more breakthroughs and advancements in the application of GPT-4 in healthcare in future studies, which could provide more effective tools for addressing the global inequality in healthcare resource distribution.

Our study is the first to explore the application of ChatGPT in the Chinese medical licensing examination. By analyzing its performance in both Chinese and English contexts, we delve into ChatGPT’s potential in medical equity. Despite this, our research still has certain limitations. The AI model we chose to represent is ChatGPT-4, which, although one of the most watched large language models at present, was not specifically trained on a medical knowledge base. Therefore, our research may underestimate the potential of AI models. Furthermore, although ChatGPT-4 has performed well in both Chinese and English versions, a licensing examination is just a qualifying test and does not fully reflect the complexity of clinical practice. Therefore, we still need to conduct more targeted specialty tests to more accurately determine the true potential of ChatGPT-4 in the field of medicine.

## Conclusion

In this study, it was found that ChatGPT demonstrates greater accuracy and response quality when answering medical questions in English compared to Chinese, highlighting a clear language bias. This bias might arise from various factors including regional policies, ambiguous question descriptions, and inadequate grasp of information. Notably, despite the superiority of the English version in both accuracy and response quality, ChatGPT displayed commendable predictiveness when dealing with questions in both Chinese and English. Furthermore, neither the difficulty nor the category of the question significantly impacted the error rate of AI’s responses.

Advanced artificial intelligence models like ChatGPT present immense potential and a promising future for global healthcare. However, to ensure their high sensitivity and accuracy across diverse linguistic and cultural backgrounds, it is imperative to address and rectify inherent language biases. This sets a direction for us to delve deeper into researching and optimizing the application of AI in the medical realm, ensuring its widespread and efficient utilization in global health scenarios.

Moreover, when confronted with localized policies and regulations, ChatGPT still has numerous challenges to overcome. The errors it exhibits in specialized fields serve as a reminder that we should not overly rely on ChatGPT. In critical situations, human review remains an indispensable step.

## Data availability statement

The raw data supporting the conclusions of this article will be made available by the authors, without undue reservation.

## Author contributions

WT, YG, and JC designed the study and established the research goals. XH was a mathematics expert and led the statistical analysis. JC, YZ, and CZ served as evaluators and rating the quality of ChatGPT 4.0’s answers. HZ provided overall support throughout the research process. All authors evaluated the answers generated by ChatGPT 4.0 and participated in the final manuscript review and approval.
