# Supplementary material for: Artificial intelligence in global health equity: an evaluation and discussion on the application of ChatGPT, in the Chinese National Medical Licensing Examination
Source: Front Med (Lausanne). 2023 Oct 19;10:1237432. doi: 10.3389/fmed.2023.1237432 (PMC10656681; doi:10.3389/fmed.2023.1237432)
Supplement: Supplementary file 1 [file Table_1.docx]

Table. Examples of response in Chinese and English Versions

| Question^a^ | Response to the Chinese Version^b^ | Average Score | Response to the English Version^b^ | Average Score |
| --- | --- | --- | --- | --- |
| Answer and explain: A. herpes simplex virus B. Coxsackie virus C. human papillomavirus  D. human immunodeficiency virus E. Cytomegalovirus 8). Male, 48 years old, recurrent blisters with itching and pain in foreskin for 1 year, good after exertion, may heal spontaneously, examination: clusters of corn-sized blisters with clear blisters are seen in foreskin. The disease is caused by ( ). | The disease is caused by A. Herpes Simplex Virus (HSV). There are two types of HSV, HSV-1 and HSV-2. HSV-2 is typically a sexually transmitted disease, primarily affecting the genitals, resulting in a sexually transmitted infection, while HSV-1 mainly affects the oral area. Both viruses can cause typical blistering lesions that may itch or hurt, usually flare up during bodily stress (such as fatigue), and tend to heal on their own. This is consistent with the symptoms of the patient you presented. Among the other options, Human Papillomavirus (HPV) typically causes genital warts, not blisters; Human Immunodeficiency Virus (HIV) leads to damage to the immune system and does not directly cause skin lesions; Coxsackie virus and Cytomegalovirus also do not cause similar symptoms. | 4 | The answer is: A. Herpes simplex virus Explanation: The description of recurrent blisters with itching and pain in the foreskin that may heal spontaneously is characteristic of genital herpes, which is most commonly caused by the Herpes Simplex Virus (HSV), specifically HSV type 2. Herpes Simplex Virus type 1 can also cause genital herpes, though it more commonly causes oral herpes. HSV is characterized by outbreaks of painful blisters or sores that come and go over time. This virus remains in the body for life, and outbreaks can recur periodically. | 4.33 |
| Answer and explain: Female, 38 years old, G2P2, contact vaginal bleeding for six months, gynecological examination, no abnormalities in vulva-vagina, cervical erosion-like, slightly raised lower lip, normal size of uterine body, no masses palpable in bilateral adnexal areas, no abnormalities in bilateral parametrium on double cochlearance, TCT examination shows HSIL, positive for high-risk HPV. 36). The most suitable surgical procedure ( ). A. extensive hysterectomy + pelvic lymph node dissection  B. cervical laminectomy  C. total extrafascial hysterectomy D. modified extensive hysterectomy + pelvic lymph node dissection E. Intrafascial total hysterectomy | The most suitable surgical method for the patient is B. Cervical conization. The patient's clinical manifestations and examination results (contact vaginal bleeding, cervical erosion, TCT indicating HSIL, high-risk HPV positive) all point to the diagnosis of High-Grade Squamous Intraepithelial Lesion (HSIL), which is a precancerous lesion of the cervix. Cervical conization is a commonly used surgery for precancerous cervical lesions, and by removing the part of the cervix containing the lesion, it can prevent the lesion from worsening and developing into cervical cancer. Other surgical methods (such as total hysterectomy and radical hysterectomy) may be overtreatment in this case, as these surgeries are more suitable for patients who have been definitively diagnosed with cervical cancer. If the cervical lesion is treated promptly and effectively, the patient may not need these larger surgeries at all. | 4.67 | The answer is: B. cervical laminectomy Explanation: This patient's primary symptom is contact vaginal bleeding, and her TCT (Thinprep Cytologic Test) examination shows HSIL (High-Grade Squamous Intraepithelial Lesion), which is a precancerous condition. Additionally, she tested positive for high-risk HPV (Human Papillomavirus), which is known to be a significant risk factor for cervical cancer. A cervical laminectomy (also known as a cone biopsy or conization) is a procedure that involves the removal of a cone-shaped piece of tissue from the cervix. This procedure is often done when there are abnormal cells in the cervix, as indicated by a pap smear or HPV test. This procedure will not only treat the lesion by removing it but also provide a specimen for pathology to assess the margins and determine if the abnormal cells have been completely removed. Hysterectomy (removal of the uterus) may be too aggressive for this situation, especially if the patient desires future fertility. It would typically be considered if invasive cancer was diagnosed, but in this case, the HSIL is a precancerous condition, not invasive cancer. Also, there's no evidence suggesting pelvic lymph node involvement requiring a lymph node dissection. However, the final treatment decision should be made based on the complete clinical picture, including further diagnostic tests if needed and the patient's preferences and overall health. | 4.67 |
| Answer and explain: 46. Male, 30 years old, one year ago, when eating in public, he began to worry about people watching him, fearing that he would make a fool of himself, so he was afraid to move his chopsticks to eat, his heart rate increased, and his hands were shaking and sweating, since then, he often avoided public meals and occasions with other people. A. Somatoform disorder B. Social anxiety disorder  C. Post-traumatic stress disorder D. Schizophrenia E. Obsessive-compulsive disorder | The patient's symptoms are most consistent with the diagnosis of B. Social Anxiety Disorder. Social Anxiety Disorder, also known as Social Phobia, is a common mental disorder characterized by excessive, irrational fear of social situations and intense worry about making a fool of themselves or being criticized. These individuals will avoid social situations that might make them uncomfortable, even though they know their fear is excessive or unreasonable. In this case, the patient experiences fear when dining in public, worries about being watched, is afraid of embarrassment, and experiences typical anxiety responses such as increased heart rate, hand tremors, and sweating. These are all typical symptoms of Social Anxiety Disorder. He avoids such situations, although he knows his fear is unnecessary, but can't control it, all of which meet the diagnostic criteria for Social Anxiety Disorder. | 4.67 | The answer is: B. Social Anxiety Disorder Explanation: Social anxiety disorder, also known as social phobia, is characterized by intense fear and anxiety of social situations where the person feels they will be judged, scrutinized, or humiliated by others. These individuals often fear that they will embarrass themselves in front of others. They might avoid social situations to a point where it interferes with their daily life and functioning. The symptoms described in the question, such as fear of public eating, worrying about people watching him, increased heart rate, shaking and sweating hands, and avoidance of public meals and social situations, are typical of social anxiety disorder. Other options like somatoform disorder, post-traumatic stress disorder, schizophrenia, and obsessive-compulsive disorder do not fit the provided clinical picture. | 4.33 |
| Answer and explain: 39. female infant, 9 months, vomiting and diarrhea for 2 days, fever for 1 day, stool 5-6 times/day, egg-flake soup-like, no odor, no pus and blood, crying with tears, decreased urine, examination: body temperature 36.5 ℃, heart rate 128 times/min, respiration 30 times/min, good spirits, flat fontanelle, skin elasticity is still possible, serving soft bowel sounds are normal, the most likely diagnosis is ( ). A. Escherichia coli infection B. Physiological diarrhea  C. rotavirus infection D. fungal diarrhea  E. Campylobacter jejuni infection | In this case, a 9-month-old female infant presents with vomiting, diarrhea (5-6 stools per day, resembling egg-flower soup), and fever. Despite her good mental state, somewhat elastic skin, and reduced urine output, the most likely diagnosis is C. Rotavirus Infection. Rotavirus is one of the main pathogens of acute gastroenteritis in infants and toddlers, with common symptoms including vomiting, diarrhea, and fever. Diarrhea caused by rotavirus is usually sudden, with stools appearing watery or egg-flower soup-like, odorless, and without obvious pus or blood. | 4.67 | The answer is: C. Rotavirus infection Explanation: Rotavirus is the most common cause of severe diarrhea among infants and young children. The symptoms described in the question, including vomiting, diarrhea, and fever, are all typical of a rotavirus infection. Rotavirus diarrhea is often watery and frequent, and it can lead to severe dehydration if not properly managed. It is also notable that the child is 9 months old, a common age for initial rotavirus infection, and the fact that the diarrhea is described as "egg-flake soup-like" is another clue, as this is often used to describe the appearance of stool in rotavirus infection. While E. coli and Campylobacter jejuni can also cause diarrhea, they are more likely to cause bloody diarrhea. Fungal diarrhea is less common and is usually seen in immunocompromised individuals or those who have been taking antibiotics. Physiological diarrhea typically does not cause fever or decrease in urination. | 4.33 |
| Answer and explain: 29. Male, 45 years old, right eyelid ptosis with diplopia for 2 months, previous history of subarachnoid hemorrhage, examination: right eye exotropia, pupil enlargement, negative reflex to light, enhanced CT suggests a 0.5 cm diameter circular high-density shadow in the saddle area, no cerebral edema. The preferred diagnosis is ( ). A. Parasellar meningioma  B. Brain abscess in the saddle area  C. Internal carotid artery-posterior communicating artery aneurysm D. Glioma of the saddle area  E. Trigeminal nerve sheath tumor | Based on the patient's history and symptoms (drooping right eyelid, double vision, outward deviation of the right eye, pupil enlargement, negative light reflex), as well as the results of an enhanced CT scan (a round high-density shadow with a diameter of 0.5cm in the saddle area, no signs of cerebral edema), the preferred diagnosis is C. Internal Carotid-Posterior Communicating Artery Aneurysm. This type of aneurysm is located at the base of the brain, and when it enlarges, it may compress nearby nerves, especially the third cranial nerve (oculomotor nerve), leading to third cranial nerve palsy, manifested as drooping eyelid, dilated pupil, disappearance of light reflex, and external strabismus. Moreover, rupture of the aneurysm can also lead to subarachnoid hemorrhage, which is consistent with the patient's previous medical history. | 5 | The answer is: C. Internal carotid artery-posterior communicating artery aneurysm Explanation: The symptoms described in the question, including ptosis (drooping of the upper eyelid), diplopia (double vision), right eye exotropia (outward turning of the eye), and a dilated pupil with a poor light reflex, suggest an impairment of the third cranial nerve (oculomotor nerve), which controls most of the eye movements and eyelid elevation. In the context of a previous subarachnoid hemorrhage, these symptoms are highly suggestive of a posterior communicating artery aneurysm. The posterior communicating artery is a small artery that connects the internal carotid artery to the cerebral arteries in the back of the brain, and an aneurysm here can press on the oculomotor nerve causing these symptoms. The other options (A, B, D, E) could potentially cause similar symptoms, but the presence of a high-density shadow in the saddle area on CT scan and the history of subarachnoid hemorrhage make the diagnosis of a carotid-posterior communicating artery aneurysm more likely. | 5 |
| Answer and explain: 10. Female, 35 years old, 22 weeks' gestation, abdominal pain for 2 weeks, aggravated by fever for 2 days. She has no vaginal bleeding and has a 5-year history of uterine fibroids. On examination: no contractions are palpable. Ultrasound shows a 10 cm solid bulge in the anterior uterine wall. The most likely diagnosis is ( ). A. Uterine leiomyoma with red discoloration B. Uterine leiomyoma with sarcoma-like changes C. fibroids combined with vitreous lesions D. fibroids combined with infection  E. Uterine fibroids combined with cystic changes | The most likely diagnosis is A. Uterine fibroids with red degeneration. Based on the case description, the patient has a 5-year history of uterine fibroids, is 22 weeks pregnant, has had abdominal pain for 2 weeks, and has been getting worse with fever for 2 days. These symptoms are consistent with the manifestations of red degeneration (red necrosis). Red degeneration refers to the acute ischemic necrosis of uterine fibroids, usually associated with pregnancy, acute torsion, or rapidly growing fibroids. Red degeneration can cause severe abdominal pain and fever. The possibilities of other options are relatively low: B. Uterine fibroids with sarcomatous degeneration - Sarcomatous degeneration is a rare malignant transformation and usually does not cause acute symptoms. C. Uterine fibroids with hyalinization - Hyalinization refers to the partial fibrosis of fibroids, usually asymptomatic. D. Uterine fibroids with infection - Although infection may cause fever, it is relatively rare in the absence of vaginal bleeding and acute onset. E. Uterine fibroids with cystic degeneration - Cystic degeneration is usually asymptomatic and does not cause abdominal pain and fever. Therefore, the most likely diagnosis is uterine fibroids with red degeneration. | 4.33 | The answer is: A. Uterine leiomyoma with red discoloration Explanation: The woman in the question has a history of uterine fibroids and is now experiencing abdominal pain and fever during her pregnancy. These symptoms, along with the ultrasound findings, suggest that one of her fibroids has undergone a process called red degeneration or necrobiosis. Red degeneration occurs when a fibroid grows too large for its blood supply, causing it to starve and die. This can lead to acute, severe abdominal pain, and it's more common during pregnancy due to increased blood flow to the uterus. Fever can sometimes occur as well. "Red discoloration" refers to the changes that occur in the fibroid as it breaks down and bleeds, which can sometimes be seen on ultrasound. Here's why the other options are less likely: B. Uterine leiomyoma with sarcoma-like changes: Sarcomatous changes would be a concern in a rapidly growing fibroid, especially in a non-pregnant woman, but it doesn't typically cause acute symptoms like the ones described in the question. C. Fibroids combined with vitreous lesions: This is not a recognized medical condition, and it's unclear what "vitreous lesions" would refer to in this context. D. Fibroids combined with infection: While infection could potentially cause fever and pain, it would be unusual for a fibroid to become infected without some type of invasive procedure having been done. E. Uterine fibroids combined with cystic changes: While fibroids can sometimes develop cystic changes, this wouldn't typically cause the acute symptoms described here. | 5 |
| Answer and explain: 14. Female, 40 years old, intermittent cough and sputum for 15 years, shortness of breath during activity for 5 years, intermittent bilateral lower limb edema for 2 years. On examination: jugular venous filling, reduced breath sounds in both lungs, heart rate 90 beats/min, rhythmical, P2 > A2, grade 2/6 systolic murmur audible in the tricuspid valve auscultation area, the most likely diagnosis is ( ). A. rheumatic heart valve disease  B. congenital heart disease with atrial septal defect C. constrictive pericarditis  D. chronic pulmonary heart disease  E. Chronic thrombotic pulmonary hypertension | Female, 40 years old, intermittent cough with phlegm for 15 years, shortness of breath during activities for 5 years, intermittent edema of both lower limbs for 2 years. Physical examination: jugular vein distention, reduced breath sounds in both lungs, heart rate 90 bpm, regular rhythm, P2＞A2, a 2/6 systolic murmur can be heard in the tricuspid valve auscultation area. The most likely diagnosis is D. Chronic cor pulmonale. Chronic cor pulmonale (pulmonary heart disease) is caused by chronic lung disease leading to pulmonary hypertension and right heart failure. Symptoms include long-term cough, phlegm, shortness of breath during activities, edema of both lower limbs, etc. Physical examination can reveal jugular vein distention, reduced breath sounds in both lungs, increased heart rate, P2＞A2 (enhanced second heart sound of the pulmonary valve), and systolic murmur in the tricuspid valve area. Among other options, rheumatic heart valve disease (A) may cause cardiac symptoms, but not long-term cough and phlegm. Congenital heart disease, atrial septal defect (B) may cause cardiac symptoms, but does not fully match the symptoms and signs described in the case. Constrictive pericarditis (C) can affect cardiac function, but not long-term cough and phlegm. Chronic thromboembolic pulmonary hypertension (E) can cause pulmonary hypertension, but does not fully match the symptoms and signs described in the case. | 5 | The correct answer is D. Chronic pulmonary heart disease. This patient has a long history of cough and sputum production, which suggests a chronic lung disease, possibly chronic obstructive pulmonary disease (COPD). Over time, chronic lung diseases can lead to pulmonary hypertension (increased pressure in the pulmonary arteries) and subsequently to right-sided heart failure. This is known as chronic pulmonary heart disease, also called cor pulmonale. The signs and symptoms described, including shortness of breath on exertion, bilateral lower limb edema (indicative of fluid retention due to right-sided heart failure), jugular venous distension (another sign of right-sided heart failure), and a louder P2 heart sound (indicative of pulmonary hypertension), all support this diagnosis. The systolic murmur heard over the tricuspid area might be due to tricuspid regurgitation, which can occur in right-sided heart failure. Here's a brief explanation of the other options: A. Rheumatic heart valve disease: This typically affects the left-sided heart valves (mitral and/or aortic) rather than causing right-sided heart failure. B. Congenital heart disease with atrial septal defect: This can lead to right-sided heart failure, but the long history of respiratory symptoms and the absence of any mention of a murmur characteristic of an atrial septal defect make this less likely. C. Constrictive pericarditis: This could potentially cause right-sided heart failure, but it wouldn't typically cause the respiratory symptoms described. E. Chronic thrombotic pulmonary hypertension: This could lead to right-sided heart failure, but it would typically cause more acute symptoms rather than the long-standing symptoms described here. Also, this condition would be more likely to cause pleuritic chest pain and hemoptysis. | 4.67 |
| Answer and explain: 45. Male, 10 years old, presented with fever and mucosal bleeding on the skin for 2 days. Hemorrhagic spots are seen on the extremities, the liver and spleen are not large, and routine blood tests: hemoglobin 75 g/L, leukocytes 1.5×109/L, platelets 20×109/L, peripheral blood 0.02 naive cells, reticulocytes 0.02. The most likely diagnosis for the child is ( ). A. Myelodysplastic syndrome  B. Aplastic anemia  C. Paroxysmal sleep hemoglobinuria D. megaloblastic anemia  E. Acute leukemia | The answer is B. Aplastic Anemia. This 10-year-old boy presented with symptoms such as fever and bleeding of the skin and mucous membranes. The results of the complete blood count showed decreased levels of hemoglobin (Hb), reduced white blood cell count, and decreased platelet count. These signs suggest the child has a problem with pancytopenia. Aplastic anemia is a disease characterized by a decline or failure of bone marrow hematopoiesis, resulting in pancytopenia, including red blood cells, white blood cells, and platelets. Patients are prone to symptoms such as anemia, infection, and bleeding. Based on the clinical manifestations and complete blood count results of the child, aplastic anemia is the most likely diagnosis. Other options do not match the child's symptoms and laboratory test results. | 4.33 | The correct answer is B. Aplastic anemia. The patient's symptoms and laboratory results suggest aplastic anemia. This condition is characterized by pancytopenia, which is a reduction in the number of all types of blood cells: red blood cells (leading to anemia, as indicated by the low hemoglobin level), white blood cells (leading to leukopenia, as indicated by the low leukocyte count), and platelets (leading to thrombocytopenia, as indicated by the low platelet count). The absence of immature cells (naive cells) in peripheral blood and low reticulocyte count also suggest a failure of the bone marrow to produce new blood cells, which is characteristic of aplastic anemia. Here's a brief explanation of the other options: A. Myelodysplastic syndrome: This group of disorders is characterized by poorly formed blood cells or cells with abnormal function due to a disruption in the production of blood cells. However, it is quite rare in children. C. Paroxysmal nocturnal hemoglobinuria: This rare disorder leads to the destruction of red blood cells, which can cause symptoms similar to those of aplastic anemia. However, it doesn't typically present with the bone marrow failure seen in this patient's laboratory results. D. Megaloblastic anemia: This type of anemia is characterized by the presence of unusually large, immature red blood cells (megaloblasts), which is not indicated in the patient's results. E. Acute leukemia: This is a type of cancer that affects the white blood cells and can lead to an increase in the number of immature white blood cells (blasts) in the blood and bone marrow, which is not seen in this case. | 5 |
| Answer and explain: Male, 48 years old, was hit by a motor vehicle on the abdomen 1 hour ago and complained of abdominal pain that gradually worsened. Examination: T 38.5℃, P 90 times/min, R 20 times/min, BP 100/74mmHg. painful appearance, intact abdominal wall, total abdominal pressure pain, rebound pain, and muscle tension, mainly around the umbilicus. Abdominal ultrasound: small amount of fluid between the intestines, no obvious abnormalities in the perihepatic and subdiaphragm. During catheterization, 300 ml of yellow urine was exported. 1). The most likely diagnosis is ( ). A. hepatic rupture B. Splenic rupture C. small intestine rupture D. ruptured pancreas E. Bladder rupture | The answer is C. Small Intestine Rupture. According to the case description, this 48-year-old man was hit by a motor vehicle in the abdomen an hour ago and his abdominal pain gradually worsened. The examination found generalized abdominal tenderness, rebound pain, and muscle tension, most pronounced around the navel. Abdominal B-mode ultrasound showed a small amount of fluid between the intestines, with no obvious abnormalities around the liver or under the diaphragm. The catheterization produced 300ml of yellow urine. Combining this information, the most likely diagnosis is a small intestine rupture. Impact injuries can cause visceral injuries in the abdominal cavity, and the small intestine is one of the areas in the abdominal cavity that is most vulnerable to damage. The patient's abdominal pain, generalized abdominal tenderness, rebound pain, and muscle tension, as well as the fluid found between the intestines on the abdominal B-mode ultrasound all support the diagnosis of a small intestine rupture. Other options such as liver rupture (A), spleen rupture (B), pancreatic rupture (D), and bladder rupture (E), although possible in abdominal impact injuries, do not align with the patient's abdominal B-mode ultrasound results showing no obvious abnormalities around the liver or under the diaphragm, and the normal yellow urine produced during catheterization. | 4.33 | The correct answer is C. Small intestine rupture. In the context of the patient's history (motor vehicle accident, abdominal pain that has gradually worsened, and signs of peritonitis like pressure pain, rebound tenderness, and muscle tension) combined with the ultrasound findings of fluid between the intestines, a rupture of the small intestine is the most likely diagnosis. A. Hepatic rupture and B. Splenic rupture: These would likely result in fluid accumulation in the perihepatic and subdiaphragmatic regions, which is not observed in the ultrasound. D. Pancreatic rupture: This is a less likely option because it's less commonly affected in blunt abdominal trauma, and it would typically present with other symptoms, such as back pain and symptoms related to a decrease in pancreatic function. E. Bladder rupture: The catheterization resulted in 300ml of yellow urine being exported. This suggests that the bladder is intact. In case of bladder rupture, you would typically see hematuria and inability to void. | 4.33 |
| Answer and explain: 10. female, 46 years old, weakness and poor appetite for 1 month, has been vegetarian, blood test: HB 74g/L, MVC 124fl, MCHC 34%, the most likely diagnosis is ( ). A. megaloblastic anemia B. Aplastic anemia C. Iron deficiency anemia  D. Marine anemia E. Acute leukemia | Female, 46 years old, loss of strength and poor appetite for 1 month, vegetarian, blood test: HB 74g/L, MVC 124fl, MCHC 34%, the most likely diagnosis is (A. Megaloblastic anemia). Explanation: The patient's hemoglobin level is reduced (HB 74g/L), suggesting anemia. At the same time, the patient's mean corpuscular volume (MVC) is increased (124fl), and the mean corpuscular hemoglobin concentration (MCHC) is normal (34%), suggesting megaloblastic anemia. The patient has been a vegetarian for a long time, which could lead to a deficiency of vitamin B12 and folic acid, leading to megaloblastic anemia. Other options: B. Aplastic anemia: Typically presents as pancytopenia with hypoplastic bone marrow, which does not match the case description. C. Iron deficiency anemia: Typically presents as microcytic hypochromic anemia, which does not match the case description. D. Thalassemia: Also known as Mediterranean anemia, typically presents as microcytic hypochromic anemia, which does not match the case description. E. Acute leukemia: Typically presents as pancytopenia with abnormal bone marrow proliferation, accompanied by systemic symptoms such as fever, bleeding, etc., which does not match the case description. | 4.67 | The patient's symptoms and lab findings suggest a diagnosis of megaloblastic anemia, specifically pernicious anemia, which is common in individuals who have a vegetarian diet and might lack vitamin B12. Megaloblastic anemia (Option A) is a condition in which the bone marrow produces unusually large, structurally abnormal, immature red blood cells (megaloblasts). The Mean Corpuscular Volume (MCV), which measures the average size of red blood cells, is typically elevated in megaloblastic anemia, as indicated by the MCV of 124fl in this case (normal range is about 80-100 fl). The MCHC (Mean Corpuscular Hemoglobin Concentration) is within normal range (roughly 31-36 g/dL). Iron deficiency anemia (Option C) usually presents with a low MCV (microcytic anemia). Aplastic anemia (Option B) involves a deficiency of all blood cell types, not just red blood cells. "Marine anemia" (Option D) appears to be a typographical error; it could potentially refer to "macrocytic anemia," but this term is less specific than "megaloblastic anemia." Acute leukemia (Option E) is a blood cancer that would typically present with an increase in white blood cells, not just anemia. Therefore, the answer should be: A. megaloblastic anemia | 5 |

Note: a: The source of the items is the 2022 National Medical Licensing Examination (NMLE); b: The content is generated by ChatGPT-4.
